# Characterization of the complete chloroplast genome of longan (*Dimocarpus longan* Lour.) using illumina paired-end sequencing

**DOI:** 10.1080/23802359.2017.1413310

**Published:** 2017-12-07

**Authors:** Kangyu Wang, Li Li, Mingzhu Zhao, Shaokun Li, Honghua Sun, Yanxi Lv, Yi Wang

**Affiliations:** College of Life Science, Jilin Agricultural University, Changchun, China

**Keywords:** Dimocarpus longan, chloroplast genome, illumina sequencing, phylogenetic analysis

## Abstract

Longan (*Dimocarpus longan* Lour.), as a kind of commercial fruit tree in the family Sapindaceae, is widely cultivated in South Asia. In this study, we obtained the complete chloroplast genome sequence of longan using Illumina paired-end sequencing. It has 160,833 bp in length, containing a pair of IR regions (28,428 bp) separated by a small single-copy region (18,270 bp) and a large single-copy region (85,707 bp). The overall GC contents of the chloroplast genome were 37.8%. This circular genome contains 130 annotated genes, including 85 protein-coding genes, 37 tRNAs and 8 rRNAs. The phylogenetic analysis using maximum–likelihood (ML) and neighbour-joining (NJ) method showed that longan has the closest relationship with *Litchi chinensis*, *Sapindus mukorossi* and *Dodonaea viscosa.* This complete chloroplast genomes can be subsequently used for the genetic breeding of this valuable species.

Longan [*Dimocarpus longan* Lour.], a tropical subtropical fruit tree in the family Sapindaceae, has great economic value in Southeast Asia. It has been cultivated in China for more than 2000 years, and during this period, 300 cultivars have been selected (Li and Zhuang 1983). Among those cultivars, only 30–40 are grown commercially (Menzel and Waite 2005). And during the production of these cultivars, a number of issues remain, including the need of improving the quality and agronomic characteristics of the fruit, which has become the key focus of the longan breeding (Lin et al. 2005). The identification and characterization of cultivar is a very important step in the breeding process, which is recently facilitated by the genome information of available resources. In higher plants breeding, many molecular markers were developed based on the sequences of chloroplast genome, contributing to the improvement of cultivar quality. However, sequences of complete chloroplast in the genus *Dimocarpus* is very limited. In this study, we obtained the complete chloroplast genome of *D. longan* and explored the phylogenetic relationship with other species, which contributes to phylogenetic studies of these taxa and better identification of different cultivars within this species.

The specimen of *D. longan* was isolated from Jilin Agricultural University test field in Changchun, Jilin, China (125.24E; 43.48N) and the DNA of *D. longan* was stored in Jilin Agricultural University College of Life Science (No. JLAUCLS2). The DNA sample was sequenced using the Illumina X-Ten Sequencing Platform (Illumina, CA). Quality control was performed to remove low-quality reads and adapters using the FastQC software (Andrews 2010). The chloroplast genome was assembled with SPAdes v3.8 (http://bioinf.spbau.ru/spades) (Bankevich et al. 2012) and annotated by DOGMA (http://dogma.ccbb.utexas.edu/) (Wyman et al. 2004). The tRNA genes were further identified using ARAGORN (Laslett and Canback 2004). The annotated chloroplast genome was submitted to GenBank database under accession No. MG214255.

The complete chloroplast genome of *D. longan* is a circle with 160,833 bp in length, containing a pair of inverted repeat regions (IRs) of 28,428 bp, a large single-copy region (LSC) of 85,707 bp and a small single-copy region (SSC) of 18,270 bp. In total, 130 genes were annotated on this chloroplast genome, including 85 protein-coding genes (PCG), 37 transfer RNA genes (tRNA) and 8 ribosomal RNA genes (rRNA). In the IR regions, a total of 19 genes were found duplicated, including eight PCG species (*rps3, rps7, rps12, rps19, rpl2, rpl22, rpl23 & ndhB*), seven tRNA species (*trnA-UGC, trnI-CAU, trnI-GAU, trnL-CAA, trnN-GUU, trnR-ACG & trnV-GAC*) and four rRNA species (*rrn4.5, rrn5, rrn16 & rrn23*). The overall nucleotide composition is: 30.7% A, 31.5% T, 19.3% C, and 18.5% G, with a total G + C content of 37.8%.

To validate the phylogenetic position of longan, the genomewide alignment of 78 plants completed chloroplast genomes was constructed by HomBlocks (https://github.com/fenghen360/HomBlocks) (Bi et al. 2017). The phylogenetic trees were reconstructed using maximum-likelihood (ML) and neighbour-joining (NJ) methods. ML analysis were performed using RaxML-8.2.4 (Stamatakis 2014), of which the bootstrap values were calculated using 1000 replicates to assess node support. NJ phylogenetic tree was constructed using MEGA7 with 1000 bootstrap replicate (Kumar et al. 2016). All the nodes were inferred with strong support by the ML and NJ methods. As shown in the phylogenetic tree (Figure 1), the chloroplast genome of longan was clustered with *Litchi chinensis, Sapindus mukorossi* and *Dodonaea viscosa.*

**Figure 1.**
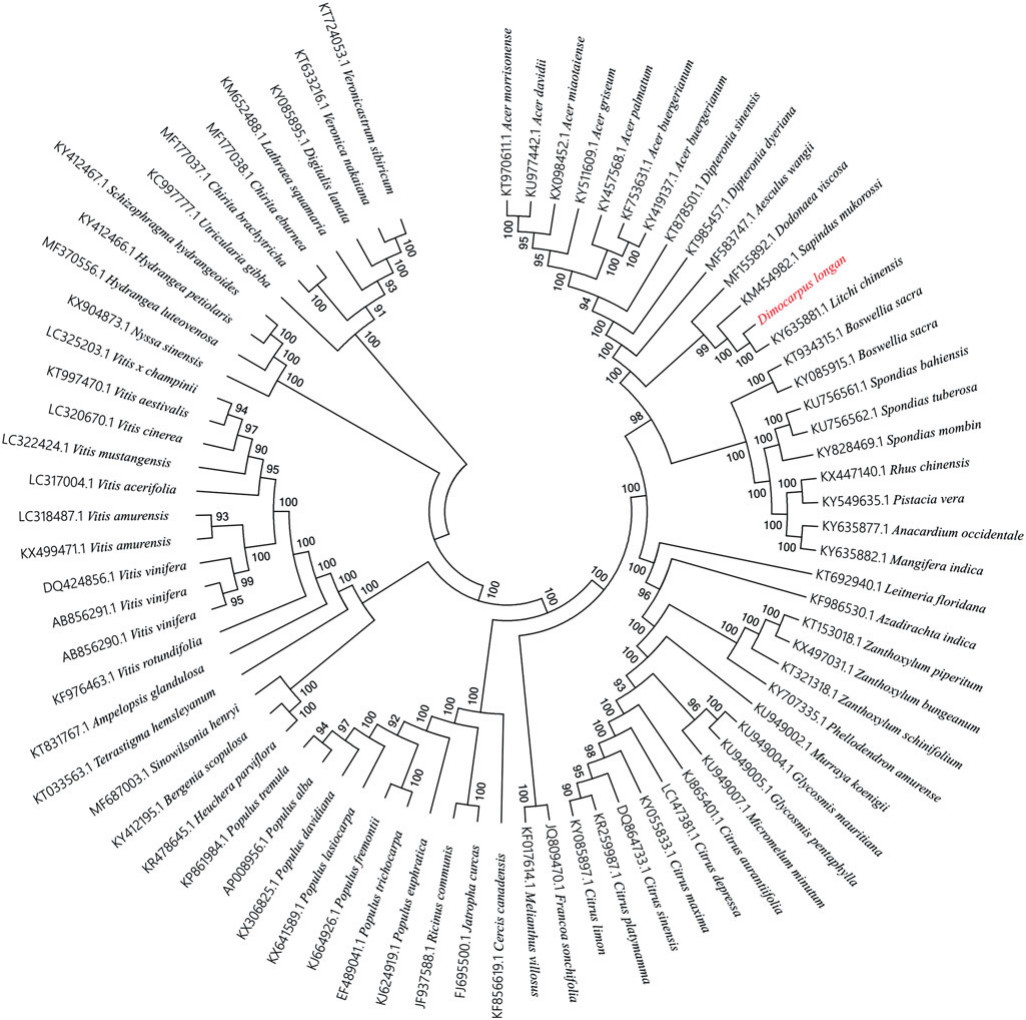
Phylogenetic relationships among 78 plant chloroplast genomes. This tree was drawn without setting of an outgroup. All nodes exhibit above 90% bootstraps. The length of branch represents the divergence distance.
